# Colchicine in stroke prevention: potential benefits and limitations of an anti-inflammatory therapy

**DOI:** 10.3389/fneur.2025.1529458

**Published:** 2025-02-13

**Authors:** Osman Corbali, Fadi Nahab

**Affiliations:** Department of Neurology, School of Medicine, Emory University, Atlanta, GA, United States

**Keywords:** colchicine, NLRP3 inflammasome, atherosclerosis, stroke prevention, inflammation, ischemic stroke, coronary artery disease

## Introduction

Inflammation has been associated with recurrent stroke risk through mechanisms involving atherosclerosis, atrial cardiopathy, blood–brain barrier dysfunction and biomarkers such as interleukin-6 (IL-6) and high-sensitivity C-reactive protein (hsCRP) which are proposed to identify patients at increased vascular risk (1).

Colchicine, traditionally used for gout and familial Mediterranean fever, has emerged as a promising cardiovascular agent. By inhibiting microtubule assembly, it has pleiotropic effects; on a cellular level it reduces endothelial dysfunction, limits smooth muscle cell proliferation, decreases macrophage adhesion, lowers platelet activation, and on a molecular level dampens the NLRP3 inflammasome pathway, reducing expression of cytokines like IL-1β and IL-6 (2, 3, 18).

In 2023, the FDA approved low-dose colchicine (0.5 mg daily) for reducing cardiovascular event risk. Despite this, it remains underutilized in neurology due to unclear benefits in stroke prevention. Could certain stroke patient subgroups benefit from colchicine?

## Clinical evidence

Ischemic stroke, a major adverse cardiovascular event (MACE), has been investigated in multiple randomized clinical trials with colchicine in patients with cardiovascular disease, yielding promising findings and trends toward reduced ischemic stroke rates (4–9). These studies included patients with chronic coronary artery disease or acute myocardial infarction (MI), with follow-up periods ranging from few months [as in studies Mewton et al. 3 months (4) and by Raju et al. 30 days (7); colchicine dose: 1 mg/day] to over 1 year [Nidorf et al. (5): 36 months (5); Nidorf et al. (6): 28.6 months (6); Tong et al. 12 months (9); Tardiff et al. 22.6 months (8); low-dose colchicine: 0.5 mg/day]. A meta-analysis by Ma et al. (10) of these placebo-controlled studies found an ~50% lower stroke rate in patients treated with colchicine (RR: 0.48; 95% CI: 0.30–0.76; *p* < 0.01). In a subgroup analysis by age, patients under 65 showed a significant reduction in stroke rate (RR: 0.35; 95% CI: 0.16–0.74; *p* < 0.01), while the reduction in patients over 65 did not reach statistical significance (RR: 0.60; 95% CI: 0.33–1.09, *p* = 0.09) (10). Similarly, Escalera et al. (11) conducted a meta-analysis involving four of these trials with low-dose colchicine (0.5 mg daily) and reported a comparable 50% reduction in stroke risk (0.43 vs. 0.88%; RR: 0.50; 95% CI: 0.31–0.82; *p* = 0.006). Among every 1,000 patients treated over 5 years, 11 strokes and 22 MIs were avoided.

Long-term Colchicine for Prevention of Vascular Events in Non-Cardioembolic Stroke (CONVINCE) was a randomized, open-label, blinded-endpoint trial that evaluated colchicine (0.5 mg daily) plus usual care vs. usual care alone in patients who had a non-cardioembolic stroke within the previous 3–28 days (12). Conducted over 7 years (2016–2022), the study enrolled 3,154 patients from over 100 hospitals in Europe and Canada but was halted early due to the COVID-19 pandemic, reaching 338 primary outcome events (initially aimed 367 events for 80% power at a 5% two-sided significance level). Patients were enrolled an average of 9 days post-stroke with a mean NIH Stroke Scale score of 1.65; 30% had lacunar strokes, and 20% had carotid stenosis. Over a median 33.6-month follow-up, the primary endpoint occurred in 9.8% of the colchicine group vs. 11.7% of controls (HR 0.84; 95% CI 0.68–1.05). Ischemic stroke was reduced by an absolute 1.7% (6.9 vs. 8.6%; HR 0.80; 95% CI 0.62–1.03), though not statistically significant, and there was no reduction in cardiac events. C-reactive protein levels were consistently lower in the colchicine group at both 28 days and annual follow-ups.

The CHANCE-3 trial [colchicine in high risk patients with acute minor-to-moderate ischemic stroke or transient ischemic attack (TIA)], a prospective randomized clinical trial, evaluated early recurrent stroke outcomes in ischemic stroke patients who received low dose colchicine for 90 days (13). It included patients with a NIHSS score of five or less or high-risk TIA) and a baseline hsCRP level of ≥2 mg/L. The trial found no difference in early recurrent stroke outcomes.

## Safety profile

Colchicine has a narrow therapeutic window, and higher doses are associated with an increased risk of side effects, particularly myopathy when used concurrently with statins. A meta-analysis showed that the incidence of myopathy among patients taking both colchicine and statins was 0.059% (461/7,779) (14). In comparison, the incidence was 0.018% in those taking statins alone (18,386/1,003,814) and 0.019% in those on colchicine alone (348/18,394). The reporting odds ratio (OR) for myopathy was 24.76 for patients taking both colchicine and statins, compared to an OR of 7.55 for colchicine alone and 11.69 for any statin alone (14). Pravastatin (OR 13.67) and lovastatin (OR 8.60) showed a trend toward lower myopathy risk when combined with colchicine, whereas atorvastatin (OR 25.73), rosuvastatin (OR 25.73), and simvastatin (OR 30.08) demonstrated similar risks when used with colchicine.

Despite this theoretical myopathy risk, clinical trials with low dose colchicine have shown an overall good safety profile. In the CHANCE-3 trial, where patients were followed for 90 days (colchicine *n* = 4,176 vs. placebo *n* = 4,167), the rate of serious adverse events was similar: 2.2% in the colchicine group compared to 2.1% in the placebo group (13). Among non-serious adverse effects, diarrhea occurred in 1.7% of patients in the colchicine group vs. 0.7% in the control group, with a hazard ratio (HR) of 2.37 (1.55 to 3.63). Abnormal liver function (defined as levels ≥3 times the normal) was observed in 0.3% of the colchicine group vs. 0.1% of the control group, with an HR of 3.99 (1.13 to 14.14). Notably, no myopathy cases were reported in CHANCE-3, even though 95.5% (7,964 patients) were on concomitant statins.

In Tardiff et al.'s study (8) (colchicine *n* = 2,330 vs. placebo *n* = 2,346) with an average follow-up of 22.6 months, the incidence of serious adverse events was similar between the colchicine and placebo groups (16.4 and 17.2%, respectively). Pneumonia was the only serious adverse event significantly higher in the colchicine group (0.9 vs. 0.4%, *p* = 0.03). There was only one case of myopathy, attributed to a high-dose statin, as the patient had only taken colchicine for 8 days, 3 months prior to the event.

In the Nidorf et al. study (6) (colchicine *n* = 2,762 vs. placebo *n* = 2,760) with an average follow-up of 28.6 months, most adverse event rates were similar. Non-cardiovascular deaths were slightly higher in the colchicine group, with an incidence rate of 0.7 events per 100 person-years compared to 0.5 events in the placebo group (HR 1.51; 95% CI, 0.99 to 2.31). Myalgia was more common in the colchicine group (384/1,811, or 21.2%) compared to the placebo group (334/1,807, or 18.5%), with an OR of 1.15 (1.01–1.31). Rhabdomyolysis occurred in one patient in the colchicine group, who made a full recovery.

In the CONVINCE trial (12), the overall rate of adverse events was similar between groups, with 69.9% in the colchicine group and 70.8% in the control group (HR 0.99; 95% CI 0.94–1.03). No muscle-related side effects were observed in the colchicine group. However, certain adverse events were significantly more common with colchicine, including loose stools or diarrhea (12.1 vs. 2.0%; HR 5.42, 95% CI 3.75–7.84) and nausea (3.4 vs. 1.4%; HR 2.42, 95% CI 1.48–3.95).

## Discussion

Colchicine is FDA-approved for atherosclerotic disease, but its stroke prevention benefits are not well established, and clinical guidance is lacking for secondary stroke prevention. For early stroke prevention, the CHANCE-3 trial (12) found no significant benefit of colchicine in reducing early recurrent stroke in high-risk patients. This suggests that treatment based on established risk factors and stroke etiology should remain the primary focus.

For long term stroke prevention, meta-analyses (10, 11) based on patients with coronary disease or myocardial infarction suggest colchicine decreases stroke risk. The CONVINCE trial (12) in non-cardioembolic stroke patients showed a non-significant trend toward reduction (8.6 vs. 6.9%) and further studies are needed.

Alongside FDA approval, the European Society of Cardiology (ESC) included colchicine in its 2021 guidelines (15) for secondary prevention of atherosclerosis. Low-dose colchicine (0.5 mg daily) may be considered, particularly if other risk factors are insufficiently controlled or if recurrent cardiovascular events occur under optimal therapy (grade of recommendation IIb, level of evidence A). This recommendation was based on the studies done with coronary disease patients or myocardial infarction (6, 8).

**Figure 1:**
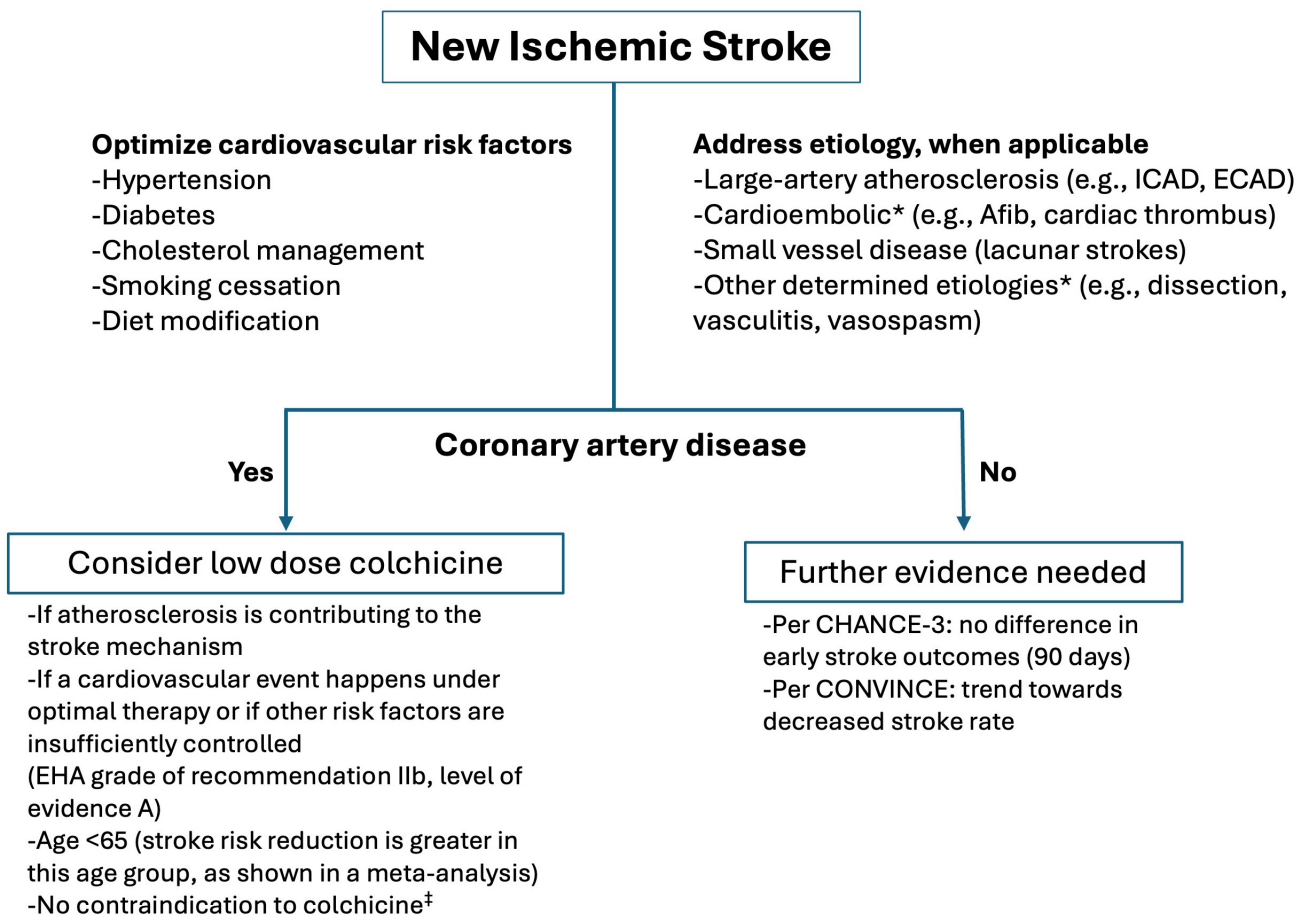
Secondary stroke prevention and colchicine. *Patients with cardioembolic stroke were excluded from both the CONVINCE and CHANCE-3 trials, and those with other determined etiology were excluded from CONVINCE. Medical conditions that raise safety concerns for colchicine therapy include renal or hepatic impairment, history of myopathy, and chronic gastrointestinal conditions such as diarrhea or inflammatory bowel disease. EHA, European Heart Association; Afib, Atrial fibrillation; ICAD, Intracranial atherosclerotic disease; ECAD, Extracranial atherosclerotic disease.

Based on the meta-analyses and ESC guidelines (15) one can consider prescribing colchicine for secondary stroke prevention if atherosclerosis is contributing to the stroke mechanism and the patient already has chronic coronary artery disease (Figure 1). Based on the meta-analyses by Ma et al. (10) younger patients ( ≤ 65 years) might benefit more from colchicine compared to older patients. Taking into consideration other risk factors and etiology of stroke, colchicine could be considered if patients have cardiovascular comorbid conditions and are having recurrent strokes/TIAs despite optimized medical therapy (Figure 1).

Low-dose colchicine can serve as an effective complementary mechanism for reducing inflammation, which plays a critical role in atherosclerotic plaque formation and rupture (16). By inhibiting the NLRP3 inflammasome and IL-1β, colchicine exerts pleiotropic effects that can be observed quickly (17), even at low doses (18). Higher doses of colchicine have been used for other conditions, such as pericarditis (0.5–1 mg), gout (0.6–1.2 mg/daily), familial Mediterranean fever (1.2–2.4 mg/daily), and COVID-19 [ranging from randomized trials using 0.5–1 mg daily doses to cohort studies employing higher loading doses of 2–4 mg; (19–21)]. Although there is limited evidence comparing dose-dependent efficacy of colchicine for cardiovascular outcomes, available data suggest that low-dose colchicine may reduce cardiovascular events more effectively than high-dose colchicine (10). Consequently, low-dose colchicine could serve as a valuable adjunct in reducing inflammation via NLRP3 inhibition. Addressing other risk factors, such as hyperlipidemia and hypertension, may further contribute to mitigating NLRP3 activation (22, 23).

While clinical trials show colchicine is generally well-tolerated, increased myopathy risk exists when combined with statins. A careful approach—prioritizing patients without hepatic or renal dysfunction and ensuring regular follow-up—may mitigate risks.

Future clinical trials, such as the Colchicine Hypertension Trial (COHERENT, NCT04916522), the Canadian Study of Arterial Inflammation in Patients with Diabetes and Vascular Events: Evaluation of Colchicine (CADENCE, NCT04181996), and the Colchicine in HFpEF study (COLPEF, NCT04857931), are expected to provide further evidence on the potential benefits of adding colchicine for stroke patients with multiple comorbidities (2). These trials may clarify colchicine's role across a broader range of comorbidities (such as hypertension, diabetes, vascular inflammation, or heart failure), informing clinical decisions in patients with complex risk profiles.

Is colchicine's benefit clinically meaningful? Ma et al.'s meta-analysis (10) found that colchicine significantly reduced stroke risk in patients with coronary artery disease or myocardial infarction (RR 0.48; 95% CI 0.30–0.76; *p* < 0.01), with 26 stroke events among 5,947 colchicine-treated patients vs. 54 among 5,919 controls. Escalera et al. (11) calculated that treating 1,000 patients over 5 years with colchicine prevents 11 strokes and 22 MIs, yielding a number needed to treat (NNT) of 90 to prevent one stroke over 5 years in CAD/MI patients. In comparison, aspirin has a NNT of 40 to prevent one recurrent nonfatal stroke over 2 years in patients with prior non-cardioembolic stroke or TIA [RR 0.81; 95% CI 0.71–0.92; incidence reduced from 130 to 105 per 1,000 patients; (24)]. Although these populations differ (CAD vs. stroke), and direct comparison is limited, colchicine's risk reduction suggests considerable preventive effects in CAD patients.

Cerebral small vessel disease (CSVD) may benefit from anti-inflammatory therapy, as age-related inflammation—mediators such as TNF, caspase-1, IL-1β, and the NLRP3 inflammasome—is considered a risk factor (25, 27). CSVD pathophysiology includes endothelial dysfunction, impaired blood-brain barrier integrity, hypoperfusion-related white matter changes, and inflammation appearing in either vascular (deep brain) or systemic (cortical) forms (25). Given colchicine's impact on certain cytokines and its anti-atherosclerotic properties, further exploration of its potential effects on CSVD is warranted (3, 25, 26).

In summary, the integration of colchicine in stroke prevention is still emerging. However, it may offer targeted benefits for select subgroups (Figure 1), and ongoing trials will further define its utility in complex cases, potentially guiding future stroke prevention strategies.
